# Validation of a German Version of the Ethical Leadership at Work Questionnaire by Kalshoven et al. (2011)

**DOI:** 10.3389/fpsyg.2016.00446

**Published:** 2016-03-31

**Authors:** Barbara Steinmann, Annika Nübold, Günter W. Maier

**Affiliations:** Work and Organizational Psychology, Department of Psychology, Bielefeld UniversityBielefeld, Germany

**Keywords:** ethical leadership, ethical leadership at work questionnaire, construct validity, criterion-related validity, nomological network

## Abstract

The present study evaluates the psychometric properties of a German version of the Ethical Leadership at Work questionnaire (ELW-D), and further embeds the construct of ethical leadership within its nomological network. Confirmatory factor analyses (CFAs) based on the total sample of *N* = 363 employees support the assumed seven-factor structure of the German translation. Within a sub-sample of *N* = 133, the ELW-D shows positive correlations with related leadership behaviors (transformational leadership, contingent reward, and servant leadership), and negative correlations with destructive ones (passive leadership, autocratic leadership, and abusive supervision), approving convergent validity of the scale. Comparisons of correlated correlation coefficients reveal restrictions of its discriminant validity. In support of the criterion-related validity (*N* = 100), the ELW-D relates to work-related attitudes (e.g., job satisfaction, satisfaction with the leader, trust in the leader) and follower behaviors (e.g., extra effort, organizational citizenship behavior) in the way expected. Besides, ELW-D-dimensions show incremental validity over and above the Ethical Leadership Scale, emphasizing the added value of this questionnaire.

## Introduction

After several scandals within the world economy, ethics has become an important topic for organizations. To be listed on New York's stock exchange, for example, companies are required by US-American law to prove their obligation to integrity guidelines. As companies suffer costly losses of reputation if they get caught in unethical conduct (Karpoff et al., 2008), and people prefer employers characterized by an ethical culture (Keith et al., 2003), organizations not only have to develop such guidelines, but also have to ensure employees behave accordingly. Evidence is accumulating that in putting integrity guidelines into practice and enhancing ethical culture and conduct of employees ethical leaders play an important role (Mayer et al., 2009; Schaubroeck et al., 2012).

Research on ethical leadership has primarily been conducted in the US where measures of business ethics are established by law. Legal requirements may affect the display and perception of these leadership behaviors just as the specific characteristics of the culture they are studied in. Although initial cross-cultural comparisons exist (Eisenbeiß, 2012; Eisenbeiß and Brodbeck, 2013), research needs to be broadened to develop a complete picture of ethical leadership and its universally endorsed or culture-specific facets. To expand cross-cultural comparisons and to promote research in Germany, a psychometrically sound German measure that thoroughly covers the construct is urgently needed. Translating the multi-dimensional Ethical Leadership at Work questionnaire (ELW) by Kalshoven et al. (2011a), we aimed to provide a comprehensive and valid multi-faceted German scale. Along with the validation, we aimed to advance the nomological network of ethical leadership. Therefore, we extended the constructs considered by Kalshoven et al. (2011a) and refined their analyses.

## Ethical leadership

Ethical leadership was first defined by Brown et al. (2005) as “the demonstration of normatively appropriate conduct through personal actions and interpersonal relationships, and the promotion of such conduct to followers through two-way communication, reinforcement, and decision-making” (p. 120). The term “normatively appropriate” is purposely kept vague as apart from universal values such as honesty, fairness, and respect, culture-specific norms and values determine the appropriateness of behavior. The definition addresses the two fundamental facets of ethical leadership: the moral person and the moral manager (Treviño et al., 2000; Brown and Treviño, 2006). As a prerequisite in leading ethically, leaders first need to be authentic moral persons (Treviño et al., 2000). The facet relates to the leader's traits, actions, and decision-making becoming apparent in his professional and personal live (Treviño et al., 2000). As moral persons, ethical leaders are upright, sincere, and fair. In making decisions they refer to solid ethical principles and consider the broader society. The moral person facet can be retrieved in other leadership styles. It overlaps with authentic, servant, transformational, or spiritual leadership (Toor and Ofori, 2009). Managing employees morally, by contrast, is unique to ethical leaders (Treviño et al., 2000; Brown and Treviño, 2006; Toor and Ofori, 2009). The facet refers to the leaders' efforts in strengthening the importance of ethics and fostering followers' ethical conduct. It is essential in bringing about the leader's reputation for leading ethically (Treviño et al., 2000; Brown and Treviño, 2006; Brown and Mitchell, 2010). Since followers emulate their leaders and return their honesty, integrity, and respect, employees' compliance and ethical conduct rise (Mayer et al., 2009). In order to be recognized as effective, ethical leaders need to be both, moral persons and moral managers.

Starting from these conceptualizations, Kalshoven et al. (2011a) sought to identify the entirety of dimensions that make up ethical leadership. They extensively reviewed research and theorizing on ethical leadership and its constituents, conducted expert interviews, and first merged the different existing concepts. In sum, they identified seven dimensions to make up ethical leadership: *people orientation, power sharing, fairness, role clarification, integrity, concern for sustainability*, and *ethical guidance.* Kalshoven et al. (2011a) found that ethical leaders respect and support followers and care about them (Treviño et al., 2003). Globally, they are characterized by high *people orientation* (Eisenbeiß and Brodbeck, 2013). They incorporate their followers' ideas and concerns in their decisions, involve them when setting performance goals, and provide them with voice (Brown et al., 2005; Kalshoven et al., 2011a). Through these means of *power sharing* they develop followers and foster their self-efficacy. As implied in the moral person facet, ethical leaders are further distinguished by their *fairness* (Treviño et al., 2000). This dimension relates to the transparent, objective, and balanced decisions ethical leaders take, but far more describes the interactions they have. Also with regard to followers' tasks, ethical leaders favor transparency. They clearly articulate their expectations, clarify responsibilities, and share the information needed to complete tasks. Since leading aims at the accomplishment of tasks (Yukl, 2012) such *role clarification* also characterizes ethical leadership (Eisenbeiß and Brodbeck, 2013). Moreover, ethical leaders act in accordance with the moral principles they preach (Brown and Treviño, 2006). They align behavior and words and keep promises, making up their *integrity* (Kalshoven et al., 2011a). They have a broad ethical awareness that exceeds beyond the organization (Treviño et al., 2003) and finds expression in a profound *concern for sustainability* (Eisenbeiß and Brodbeck, 2013; Frisch and Huppenbauer, 2013). Among others, they care about the environment and promote eco-friendly work processes (Kalshoven et al., 2011a; Frisch and Huppenbauer, 2013). Finally, ethical leaders explain values and ethics guidelines to their followers and communicate the importance of ethical standards, thereby making ethics an explicit topic on their leadership agenda (Treviño et al., 2000; Brown and Treviño, 2006). To ensure ethical standards are kept, they reward those acting in accordance, and punish those who break the rules. Through this *ethical guidance* leaders develop followers' ethical awareness. While people orientation, fairness, and integrity build the moral person facet of ethical leadership, power sharing, role clarification, ethical guidance, and concern for sustainability form the moral management facet.

Ethical leadership is associated with various positive consequences for employees and organizations. Through their moral management, ethical leaders reduce deviance and counterproductive behavior within teams and foster the ethical conduct of immediate followers (Mayer et al., 2009; Den Hartog and Belschak, 2012) as well as across hierarchical levels (Schaubroeck et al., 2012). Concurrently, they enhance the task performance of their work-group (Piccolo et al., 2010). Ethical leadership also relates to followers' engagement, initiative, commitment, and job satisfaction, their helping behavior, courtesy, and well-being, as well as to the amount of leader-member exchange and leader effectiveness followers' perceive (Neubert et al., 2009; Den Hartog and Belschak, 2012; Kalshoven and Boon, 2012; Kalshoven et al., 2012, 2013; Hassan et al., 2013). It is hence reasonable that ethical leaders are said to have a higher potential for obtaining upper management positions (Rubin et al., 2010). Though, Stouten et al. (2013) found that high levels of ethical leadership may decrease employees' voluntary behaviors.

## Measuring ethical leadership

### The ELS

Along with their definition, Brown et al. (2005) developed the first measurement instrument of ethical leadership, the Ethical Leadership Scale (ELS). The ELS has widely been used to study the phenomenon, but has lately been criticized for theoretical as well as practical reasons, and limitations of the scale have been discussed: First, the wording of the items remains vague (Frisch and Huppenbauer, 2013). Although Brown et al. (2005) emphasized the importance of visible actions to be recognized as ethical leader, the ELS does not sufficiently specify the behaviors associated with ethical leadership (Tanner et al., 2010). Among others, participants are asked whether their leaders conduct their lives in an ethical way. Such items require an understanding of ethics on the part of the respondents which is not self-evident—a second limitation of the ELS (Tanner et al., 2010). Third, the compilation of items has been questioned. Items that refer to considerate instead of ethical leader behaviors are included, whereas core facets like integrity, power sharing, or certain fairness aspects are missing (Yukl et al., 2013). Fourth, constituting behaviors and consequences have been intermingled. Trust in the leader is considered a feature of ethical leadership, although it rather results from such behaviors (Tanner et al., 2010). Fifth, the ELS solely regards the leader's behaviors toward employees, no further stakeholders are considered (see Frisch and Huppenbauer, 2013). Not least, tests of discriminant validity were partly flawed by the wording of the items used to determine validity (Yukl et al., 2013). Besides, the ELS highly correlates with transformational leadership (Brown et al., 2005) questioning its discriminant validity.

### The ELW

As the ELS does not exhaustively cover the behaviors revealed by Kalshoven et al. (2011a), the authors developed and validated a new questionnaire. The multi-dimensional ELW continues and refines the ELS (Eisenbeiß, 2012) and faces several of its limitations. Refinements mainly concern the wording and the compilation of items. ELW-items were adapted from earlier research, resulted from interviews with managers and employees or were self-developed. In generating the items, Kalshoven et al. (2011a) followed an empirical-descriptive approach, and concentrated on *concrete* ethical leader *behaviors* at work and on *actions* through which ethical leadership becomes apparent. Given the strict focus on behavioral manifestations, no competence about ethics is needed in completing the ELW. Instead of judging the ethicality of a leader's conduct, respondents rate how frequently the leader displays certain behaviors. Based on that evaluation, the degree of ethical leadership a leader exerts is then derived. In so doing, the ELW does not solely focus on the leader, but explicitly captures the way leaders and followers *interact*. Evaluating leader-follower-interactions is crucial as the ascription of ethical leadership essentially depends on the way followers perceive their leader's treatment.

Further refinements concern the compilation of items. While ELS-items stress the moral person facet and the leader's ethical role modeling, the ELW deeper assesses the leader's moral management. Since power sharing, role clarification, and concern for sustainability turned out to cross-culturally define ethical leaders (Eisenbeiß and Brodbeck, 2013), including items on these dimensions contributes to specifying the measurement. Incorporating the latter dimension, the ELW relates to at least one further stakeholder apart from followers. The focus on employees and the environment is in line with research on corporate social responsibility (CSR), which broadly corresponds to business ethics. In CSR-models, too, the environment is regarded the main stakeholder apart from people (Zwetsloot, 2003). Regarding the moral person facet, items on a leader's integrity have been included, while those on trustworthiness did not enter the scale. Moreover, instead of narrowing fairness to the decisions ethical leaders take, the ELW broadens this dimension to work-related interactions and the leader's goal striving. As such, the ELW includes core dimensions of ethical leadership not being covered by the ELS, and overcomes several of its conceptual shortcomings. In sum, the ELW represents a precise and more comprehensive measure of specific ethical leadership behaviors.

Also for practical reasons the ELW represents an improvement. The multi-dimensional ELW enables in-depth measurement of ethical leadership dimensions. From a scientific perspective, the multi-dimensionality facilitates detailed examination of the nomological network. Drawing on dimensions, relations with constructs of the same level of specification may be examined and differential relations with these constructs may be established, locating ethical leadership more clearly in a fine-grained nomological network. From a practitioner's perspective, such detailed measurement can be applied to generate ethical leadership profiles based on which individually tailored leadership trainings may be developed.

Results of the validation attest the 38-item ELW to be a reliable and valid measure of ethical leadership (Kalshoven et al., 2011a). Confirmatory factor analyses (CFAs) confirmed the assumed seven-factor structure of the scale. Together these dimensions load on a higher-order ethical-leadership-factor (Kalshoven et al., 2011a, 2013). The ELW relates positively to related and negatively to destructive leader behaviors. ELW-dimensions are positively associated with followers' work attitudes and perceived leader effectiveness.

## The present study

The aim of the present study was to translate the ELW into German (ELW-D), to validate the German scale, and to show accordance of ELW-D and ELW. Therefore, we drew on the constructs Kalshoven et al. (2011a) used to validate the ELW (transformational, passive, and autocratic leadership, as well as contingent reward to establish construct validity; and job satisfaction, satisfaction with the leader, effectiveness of the leader, organizational commitment, team commitment, trust in the organization, trust in the leader, employee effectiveness, organizational citizenship behavior (OCB), and cynicism to assess criterion-related validity). Additionally, we regarded the association with servant and abusive leadership and compared the correlation between the ELW-D and the German ELS (ELS-D; Rowold et al., 2009) with the association between the ELW-D and related leadership constructs to determine convergent and discriminant validity. Besides, we examined the impact ethical leaders have on psychosocial processes within teams (perceived psychological safety and group cohesiveness), work-related aspects (job clarity), and followers' motivation (extra effort). Analyses were led by the following assumptions:

### Factor structure

The ELW-D consists of the seven interrelated dimensions people orientation, power sharing, fairness, role clarification, integrity, concern for sustainability, and ethical guidance. Together these dimensions constitute an overarching ethical-leadership-factor.

### Convergent and discriminant validity

As ethical leadership shares certain characteristics with transformational leadership (showing concern for others, exerting idealized influence, being a role model, aligning values) and contingent reward (clarifying roles, rewarding required conduct and performance, setting standards), we assume substantial correlations between these constructs to indicate convergent validity of the ELW-D. Extending beyond Kalshoven et al. (2011a), we suppose that ethical and servant leaders also share characteristics (being upright, empowering followers, exceeding beyond self-interests, showing consideration). High positive correlations between these constructs further support convergent validity. Besides, we examined the association between ethical leadership and several destructive leader behaviors. Destructive leader behaviors are central to unethical leadership, and thus represent the construct domain of ethical leadership (Brown and Mitchell, 2010). Since destructive leaders show behaviors opposed to those of ethical ones, high negative relations between measures of these constructs provide evidence for convergent validity. In accordance with Kalshoven et al. (2011a), we analyzed the relation with passive and autocratic leadership. Leader-follower interactions of such leaders are characterized by indifference, non-participation, and non-nurturance, contradicting the participation, consideration, and guidance ethical leaders provide. We hence assume high negative associations between ethical and passive as well as autocratic leadership. Broadening the analyses, we additionally examined the relation with abusive leadership. Abusive leaders are characterized by their self-sacrifice, hostility, abusiveness, and humiliation. We expect high negative associations between abusive leadership and the ELW-D to further confirm convergent validity.

Despite its high correlations with other leader behaviors we expect ethical leadership to be a distinct construct. To examine this assumption, we compared the strength of the associations between the various leadership measures. Given that ELW-D and ELS-D are operationalizations of the same construct, these measures ought to correlate very highly. If this correlation exceeds those between the ELW-D and any of the other related, but distinct leadership measures, discriminant validity is supported.

### Criterion-related validity

With regard to criterion-related validity we expect concurrent relations with employees' work-related attitudes (job satisfaction, satisfaction with the leader, organizational commitment, team commitment, trust in the leader, trust in the organization, and cynicism) and behaviors (effectiveness and OCB), as well as their evaluation of the leader (effectiveness of the leader). Except for cynicism, these correlations are assumed to be positive. Besides, we suppose that ELW-D-dimensions account for variance increments in these outcomes over and above the ELS-D for including outcome-related ethical leadership behaviors which the ELS-D does not consider to the same extent. For instance, the ELW-D extensively measures integrity, power sharing, and fairness which foster followers' trust in the leader. As the ELS-D—if at all—only superficially taps these behaviors, ELW-D-dimensions likely account for variance increments beyond the ELS-D.

Finally, we extended the criterion domain of ethical leadership. Ethical leaders are considerate of followers, empower them, and request their integrity and fairness. That way, they create an atmosphere of respect, trust, and sincerity within teams. Faithful interactions with the leader but also among team members likely evolve. We expect that the ELW-D-dimensions people orientation, power sharing, fairness, integrity, and ethical guidance explain variance in the psychological safety and group cohesiveness within teams over and above the ELS-D. Since ethical leaders set clear standards and clarify expectations, responsibilities, performance goals, and ultimately followers' jobs, we expect role clarification and ethical guidance to account for variance increments in job clarity. Finally, we assume that employees of ethical leaders show extra effort paying back the respect they receive. If followers are granted autonomy and know their job roles and tasks, they likely show extra effort. Therefore, we expect power sharing and role clarification to account for variance increments over and above the ELS-D.

## Method

### Procedure

Translating the English items published by Kalshoven et al. (2011a) into German, we followed the guidelines by Brislin (1980). Two bilinguals translated and retranslated the 38 items. Differences concerning the wording were discussed and translators agreed upon a functionally equivalent German version (see Appendix in Supplementary Material). We were notarially granted permission to publish and use the German version of the ELW for scientific purposes by the first author of the original scale. Before starting data collection, we consulted the Ethics Committee of Bielefeld university and answered its basic application questionnaire in order to evaluate whether the study complied with ethical standards. As we did not imply any method that deviated from legal regulations or ethical guidelines, no further steps were needed to ensure ethical innocuousness of the study. Consequently, this study was carried out in accordance with the recommendations of the German Psychological Society and the Association of German Professional Psychologists as well as the German data privacy act.

To test the psychometric properties of the ELW-D, we conducted three online surveys each assessing ethical leadership and participants' demographics. While CFAs relied on the data of all surveys (*N* = 363), one of the surveys (sub-sample 1; *N* = 133) was designed to examine convergent and discriminant validity of the ELW-D. In addition to ethical leadership, followers' ratings of the various leader behaviors were gathered in this survey. We grouped positive and destructive leadership behaviors into several blocks. In rating their leaders, employees were alternately faced with these blocks, in which items were presented randomly. Another survey served to determine criterion-related validity of the ELW-D (sub-sample 2; *N* = 100). Apart from ethical leadership, we here assessed all outcome variables. Outcomes were arranged thematically, and items presented randomly within the units. In order to recruit participants for all three surveys, we approached employees in virtual business networks and other social media sites and drew on the snowball procedure. A convenient sample of employees working either full-time or part-time resulted.

In order to ensure anonymity, we did not gather participants' written informed consent to take part in the study. Yet, we emphasized that by closing the internet browser participants could abandon the survey at any time. Participants were assured that incomplete data would be deleted and would not enter the analyses, and that their anonymity would be kept. Apart from ensuring data privacy, protecting respondents' anonymity and that way reducing participants' evaluation apprehension is one of the procedural remedies suggested in controlling for common method bias (Podsakoff et al., 2003). For the same reason, participants were instructed that no right or wrong answers existed, and were asked to rate the items as honestly as possible. Further diminishing threats of common method bias, we drew on well-established and psychometrically sound measures made up of simple and specific items in assessing leadership behaviors and all outcomes except employees' effectiveness (see Podsakoff et al., 2012). Not least, randomization of items, inclusion of reverse-coded items, and the alternating presentation of positive and destructive leadership behaviors equally served the purpose of controlling for common method bias (see Podsakoff et al., 2012).

### Samples

In the overall sample, 55.6% of the participants were females, and 44.4% were males. The average age was 35.80 years (*SD* = 11.86). Half of the sample (50.3%) held university or polytechnic degrees and 35.1% had completed vocational trainings. Jobs were distributed throughout all industrial sectors. Participants had been employed in their current organizations for 8.55 years (*SD* = 9.43). Nearly one fifth (17.6%) of the participants was in a leading position.

While sub-sample 1 consisted of 42.9% females and 57.1% males, in sub-sample 2 46% were women and 54% were men. Participants' age was, on average, 38.90 years (*SD* = 11.63) in sub-sample 1, and 29.50 years (*SD* = 7.91) in sub-sample 2. In sub-sample 1, 54.1% of the employees held university and polytechnic degrees, 33.1% underwent vocational trainings. In sub-sample 2, 40% held university and polytechnic degrees and 38% completed vocational trainings. In organizations of various industrial sectors tenure was about 9 years in sub-sample 1 (*M* = 9.20, *SD* = 9.72) and 5 years and 7 months (*M* = 5.60; *SD* = 6.13) in sub-sample 2. Of these participants 8% respectively 17% had managerial responsibility.

### Measures

In the overall sample, we assessed ethical leadership in two ways: Apart from the ELW-D (38 items, α = 0.96 for the composite; people orientation: 7 items, α = 0.93; power sharing: 6 items, α = 0.80; fairness: 6 items, α = 0.93; role clarification: 5 items, α = 0.88; integrity: 4 items, α = 0.93; concern for sustainability: 3 items, α = 0.77; ethical guidance: 7 items, α = 0.90), all participants rated the leaders' ethical leadership using the ELS-D (Rowold et al., 2009; 10 items, α = 0.88).

#### Measures of additional leadership behaviors

To test construct-related validity, participants in sub-sample 1 additionally evaluated their supervisors on further leader behaviors. Measuring *transformational leadership* (20 items, α = 0.94), *passive leadership* (4 items, α = 0.80), and *contingent reward* (4 items, α = 0.75) we used the respective scales of the German Multifactor Leadership Questionnaire (MLQ; Felfe, 2006). *Servant leadership* was measured with the short form of the Servant Leadership Survey (21 items, α = 0.95, Van Dierendonck and Nuijten, 2010; Asag-Gau and van Dierendonck, 2011), *abusive leadership* with the German version of the Abusive Supervision Scale (Loock and Schilling, 2010; 10 items, α = 0.88). In rating *autocratic leadership*, participants answered the respective subscale of the German GLOBE Leadership Scale (Brodbeck and Frese, 2007; 6 items, α = 0.94). On a five-point response scale (ranging from 1 = *strongly disagree* to 5 = *strongly agree*) participants indicated how much they agreed to the statements when regarding their own leaders.

#### Measures of outcome variables

To establish criterion-related validity, in addition to ethical leadership different work-related attitudes and behaviors of employees were considered in sub-sample 2. To assess *job satisfaction* participants indicated how satisfied they were with their jobs, taking tasks, working conditions, colleagues, working hours, and the like into account (Neuberger and Allerbeck, 1978). *Satisfaction with the leader* (2 items, α = 0.83) and *effectiveness of the leader* (4 items, α = 0.85) were rated on the corresponding scales of the German MLQ (Felfe, 2006), while *organizational commitment* was assessed using the short form of the German Organizational Commitment Questionnaire (Maier and Woschée, 2002; 9 items, α = 0.94). For the assessment of *team commitment*, we applied the respective scale of the measure COMMIT (Felfe and Franke, 2012; 5 items, α = 0.87). Data on *trust in the organization* (9 items, α = 0.93) and *trust in the leader* (9 items, α = 0.93) were gathered with the German Workplace Trust Survey (Lehmann-Willenbrock and Kauffeld, 2010). *Employee effectiveness* was assessed with two questions used by Kalshoven et al. (2011a; α = 0.51) adapted to self-reports, and *OCB* with a German measure of performance-related work attitudes (Staufenbiel and Hartz, 2000; 20 items, α = 0.79). To measure *cynicism*, we used the Employee Cynicism Scale (Cole et al., 2006; 7 items, α = 0.86).

Extending the criterion domain of ethical leadership, we determined the *psychological safety* within teams by adapting the scale by Baer and Frese (2003; 7 items, α = 0.82) to the team level. *Team cohesiveness* was measured with the corresponding scale of a teamwork questionnaire (Kauffeld, 2004; 8 items, α = 0.88). To quantify *job clarity* we used the German version of the questionnaire for measuring facets of job ambiguity (Schmidt and Hollmann, 1998; 9 items, α = 0.92). Employees' *extra effort* was assessed with the respective scale of the German MLQ (Felfe, 2006; 3 items, α = 0.91).

Trust measures were rated on a six-point, OCB on a seven-point response scale (ranging from *strongly disagree* to *strongly agree*). Items on team cohesiveness were presented as five-point semantic differentials. The remaining outcomes were rated on the same five-point basis as leader behaviors.

## Results

### Factor structure

In accordance with Kalshoven et al. (2011a) we tested the goodness of fit of several models, but assumed a structure with seven correlated factors to fit the data best. Following the original work, we aggregated factors for theoretical reasons and compared one-, two-, three-, four-, five-, and seven-factor solutions using AMOS 22 (Arbuckle, 2013). To evaluate the model fit we considered the χ^2^-value divided by the degrees of freedom (χ^2^/*df*), the Root Mean Square Error of Approximation (RMSEA), the Tucker-Lewis Index (TLI), the Comparative Fit Index (CFI), and the Standardized Root Mean Square Residual (SRMR). For an acceptable fit the χ^2^/*df* -ratio lies between 2 and 3, the RMSEA between 0.05 and 0.08, TLI and CFI are >0.90 and the SRMR lies below 0.10 (see Kline, 2005). Among the models regarded, χ^2^-difference tests pointed to the seven-factor solution to be the best fitting model. The model showed an acceptable fit to the data (Table 1). Implied and observed correlations among the seven factors are presented in Table 2. Factor loadings ranged between 0.51 and 0.91 for people orientation (0.76–0.86), power sharing (0.51–0.74), fairness (0.64–0.88), role clarification (0.68–0.87), integrity (0.85–0.91), concern for sustainability (0.56–0.85), and ethical guidance (0.65–0.85, all *p* < 0.001). To examine the factor structure of the ELW-D more closely, we further assessed whether factor loadings varied across the different sub-samples. Apart from sub-samples 1 and 2, we included the remaining participants of the overall sample as a third sub-sample into our analyses. Following Byrne (2010), we first established a baseline model in each of the three sub-samples and tested whether the same number of factors was present across the samples. In each sub-sample a seven factor solution provided the best fitting model. Having shown equivalence of the number of factors, we went on testing the invariance of the factor loadings. A multigroup model with seven factors and unconstrained factor loadings was specified against which a model was compared, in which equality constraints had been imposed on the factor loadings. Invariance can be assumed if the difference in CFI between these models is <0.01 (Cheung and Rensvold, 2002). Analyses yielded a value of ΔCFI = 0.005, emphasizing invariance of factor loadings across the sub-samples.

**Table 1:** **Model fit and model comparisons of the alternative models**.

**Model**	**χ^2^ (*df*)**	**χ^2^/*df***	**RMSEA**	**TLI**	**CFI**	**SRMR**	**AIC**	**Nested model comparison**	
								***Δχ*^2^**	**Δ *df***
7 factor model	1504.48^***^	2.34	0.061	0.901	0.909	0.064	1698.475		
	(644)								
5 factor model	2132.04^***^	3.25	0.079	0.833	0.844	0.075	2302.043	627.56	12
(PO, PS and F)^a^	(656)							*p* = 0.000	
4 factor model	2433.99^***^	3.69	0.086	0.800	0.812	0.073	2597.994	929.51	15
(PO and PS) (I and F) (EG and RC)	(659)							*p* = 0.000	
3 factor model	3181.28^***^	4.81	0.103	0.717	0.734	0.089	3339.275	1676.80	18
(PO, PS and F) (I, RC and EG)	(662)							*p* = 0.000	
2 factor model	3821.38^***^	5.76	0.115	0.647	0.666	0.096	3975.375	2316.90	20
(PO, PS, F, I, EG and RC)	(664)							*p* = 0.000	
1 factor model	3920.43^***^	5.90	0.116	0.636	0.656	0.097	4072.426	2415.95	21
(uni-dimensional model)	(665)							*p* = 0.000	
2nd order ELW-D-factor model	1648.43^***^	2.51	0.064	0.888	0.895	0.077	1814.433		
	(658)								

*N = 363. PO: people orientation; PS: power sharing; F: fairness; I: integrity; EG: ethical guidance; RC: role clarification*.

a *Factors in brackets constitute one factor*.

*** *p < 0.001*.

**Table 2:** **Implied and observed correlations between the seven ELW-D-dimensions**.

	**(1)**	**(2)**	**(3)**	**(4)**	**(5)**	**(6)**	**(7)**
(1) PO		0.73	0.64	0.58	0.69	0.56	0.60
(2) PS	0.84		0.56	0.53	0.58	0.45	0.55
(3) F	0.67	0.63		0.36	0.64	0.37	0.36
(4) RC	0.62	0.60	0.37		0.58	0.43	0.68
(5) I	0.75	0.66	0.68	0.60		0.49	0.54
(6) CFS	0.73	0.64	0.51	0.53	0.66		0.57
(7) EG	0.64	0.64	0.39	0.75	0.59	0.68	

*N = 363. PO: people orientation; PS: power sharing; F: fairness; RC: role clarification; I: integrity; CFS: concern for sustainability; EG: ethical guidance. Coefficients below the diagonal display implied correlations between latent factors. Above the diagonal observed correlations are displayed*.

*All correlations are significant at p = 0.000*.

Additionally, we analyzed the profile of the interrelations among the seven ELW-D-dimensions to examine whether they were similar to those within the ELW. We correlated the coefficients of the observed correlations between one ELW-D-dimension and the other six dimensions with the same coefficients within the ELW (vector correlations; Hofmann and Jones, 2005). The resulting correlation coefficient quantifies the degree of similarity of the correlations between one dimension and the others within ELW-D and ELW. Analyses revealed the lowest coefficient for fairness. Similarity of the association pattern was still considerable (*r* = 0.89, *p* < 0.01). Relations between the dimension concern for sustainability and the other six dimensions were virtually identical within ELW-D and ELW (*r* = 1.00, *p* < 0.001). The remaining coefficients ranged between these values, indicating that interrelations are congruent among ELW-D and ELW.

In line with Kalshoven et al. (2011a), we then tested a model with an overarching ethical-leadership-factor. Whereas, χ^2^/*df*, RMSEA and SRMR attested this model an acceptable fit, TLI and CFI were below the cut-offs (Table 1). To contrast this model with the seven-factor solution, we used the Akaike Information Criterion (AIC) which compares the fit of models that need not be nested. The model with the lower AIC-value can be regarded as the better fitting model (Schermelleh-Engel et al., 2003). As the seven-factor model showed a lower AIC than the model with the overarching ethical-leadership-factor (Table 1), the seven-factor solution seems to better fit the data.

### Convergent and discriminant validity

We next considered convergent and discriminant validity of the ELW-D. As expected, correlations between ELW-D, ELS-D, transformational leadership, and contingent reward were positive and significant (Table 3). The correlation with the additionally considered construct of servant leadership was also positive and significant. In accordance with Kalshoven et al. (2011a), the ELW-D showed significant negative correlations with passive leadership. While scattered findings of non-significance were reported for the dimensions of the original, in our study there was a significant negative association between the ELW-D and autocratic leadership. Corroborating our assumptions and strengthening convergent validity, abusive leadership also significantly and negatively related to ethical leadership.

**Table 3:** **Correlation matrix of study variables in sub-sample 1**.

	***M***	***SD***	**(1)**	**(2)**	**(3)**	**(4)**	**(5)**	**(6)**	**(7)**	**(8)**	**(9)**	**(10)**	**(11)**	**(12)**	**(13)**	**(14)**
(1) PO	3.44	0.99														
(2) PS	3.48	0.78	0.76													
(3) F	4.05	0.84	0.73	0.57												
(4) RC	3.64	0.88	0.59	0.58	0.32											
(5) I	3.91	0.91	0.66	0.57	0.62	0.53										
(6) CFS	3.01	0.99	0.60	0.51	0.44	0.41	0.49									
(7) EG	3.19	0.84	0.65	0.60	0.36	0.73	0.49	0.58								
(8) ELW-D	3.54	0.71	0.92	0.84	0.74	0.75	0.77	0.70	0.81							
(9) ELS-D	3.23	0.74	0.86	0.81	0.66	0.60	0.63	0.63	0.74	0.90						
(10) TL	3.40	0.76	0.81	0.69	0.58	0.73	0.65	0.58	0.70	0.86	0.80					
(11) PL	2.36	0.74	−0.59	−0.52	−0.50	−0.65	−0.75	−0.41	−0.50	−0.70	−0.54	−0.69				
(12) CR	3.55	0.86	0.69	0.59	0.48	0.73	0.59	0.49	0.59	0.75	0.64	0.85	−0.63			
(13) SL	3.46	0.77	0.85	0.80	0.71	0.60	0.70	0.59	0.67	0.90	0.85	0.81	−0.62	0.67		
(14) AbL	1.46	0.56	−0.64	−0.47	−0.80	−0.32	−0.65	−0.45	−0.37	−0.67	−0.59	−0.62	0.53	−0.53	−0.70	
(15) Auto	1.87	0.97	−0.73	−0.66	−0.77	−0.32	−0.58	−0.50	−0.43	−0.73	−0.71	−0.62	0.45	−0.47	−0.76	0.73

*N = 133. PO: people orientation; PS: power sharing; F: fairness; RC: role clarification; I: integrity; CFS: concern for sustainability; EG: ethical guidance; ELW-D: Ethical leadership at Work questionnaire German version; ELS-D: Ethical Leadership Scale German version; TL: transformational leadership; PL: passive leadership; CR: contingent reward; SL: servant leadership; AbL: abusive leadership; Auto: autocratic leadership*.

*All correlations are significant at p = 0.000*.

Using the procedure by Meng et al. (1992), we then tested discriminant validity and analyzed whether the ELW-D correlated more highly with the ELS-D than with any of the other leadership measures. As the ELS-D is a different measure of the same construct, correlations between ELW-D and ELS-D ought to exceed those between ELW-D and any of the other measures capturing destructive or related, but distinct leader behaviors. Based on Fisher's *z* transformations of the correlation coefficients the procedure by Meng and colleagues statistically tests the difference between two correlated correlation coefficients. The ELW-D indeed displayed a higher correlation with the ELS-D than with any of the destructive leader behaviors (passive: *z* = 16.85, abusive: *z* = 16.21, autocratic: *z* = 16.41) or contingent reward (*z* = 4.84, all *p* < 0.001). However, it only marginally related more closely to the ELS-D than to transformational leadership (*z* = 1.89, *p* < 0.10). The association between the two measures of ethical leadership was not stronger than the one between ELW-D and servant leadership (*z* = 0.12, *p* > 0.05). These findings restrict the discriminant validity of the ELW-D.

### Additional analyses related to the convergent and discriminant validity of the ELW-D

Further refining the analyses of convergent validity, we also regarded the associations between the different leadership styles on the level of latent variables. In doing so, we followed Fornell's and Larcker's (1981) recommendations in determining convergent and discriminant validity in structural equation models. According to the authors, convergent validity of constructs may be assumed if the variance in indicators captured by a construct is >50%, hence exceeding the variance due to measurement error. Modeling the relations between an overarching ethical-leadership-factor and autocratic, abusive, passive, servant, and transformational leadership as well as contingent reward each, we found more than 60% of the variance (ranging between 0.63 and 0.64) in ethical leadership to be explained by the underlying construct, thus supporting convergent validity of the ELW-D. Since we assumed the various leadership concepts to overlap, constructs are assumed to also exert an influence on those observed variables that are focal indicators of the other highly related leadership construct. As such an influence indicates a lack of discriminant validity tests ought to question the discriminant validity between these constructs in order to support convergent validity. According to Fornell and Larcker (1981) two constructs are discriminable if the average variance extracted for each construct exceeds the size of the squared correlation between these constructs. For all leadership constructs apart from abusive leadership the average extracted variance of the ELW-D, the other leadership construct, or both was smaller than the squared correlation between the constructs, lending further support to our assumption of convergent validity.

Given the limitations we found with regard to the discriminant validity of the ELW-D when comparing correlated correlation coefficients, further analyses have been conducted to ensure the validity of our conclusions. As all leadership constructs have been assessed among the same participants, at the same point in time, within one questionnaire, correlations between ethical, transformational, and servant leadership may have been distorted by common method variance irrespective of the procedural remedies applied (see Podsakoff et al., 2003, 2012). In determining whether common method variance was a problem in our data, and if so, how much of the variance explained in the constructs may be attributed to a common method factor, we drew on the statistical remedies recommended by Podsakoff et al. (2003, 2012). Since no marker variable was present in our data, we controlled for the impact of a single unmeasured latent method factor. Given the complexity of the model, we were unable to include all leadership variables into a single CFA, but instead conducted two analyses. We entered contingent reward, passive, abusive, autocratic, and ethical leadership in one of the analyses, while the second analysis checked for common method variance across ethical, transformational, and servant leadership. In either case, we first ran the CFAs without the common latent factor. In a second step, we included a common latent factor into our analyses, whose effect was modeled on each of the indicators. In order to determine the amount of variance attributable to the common method latent factor, in a final model, regression weights of the latent factor were constrained to be equal. In the model considering contingent reward, passive, abusive, autocratic, and ethical leadership, common method variance did not seem to affect the analyses: Only 4% of the variance resulted from common method variance. Moreover, we compared the size of the correlations among the seven ELW-D-dimensions as well as those between ELW-D-dimensions and the other four leadership constructs measured in the model without a common method factor, with the corresponding correlations obtained from the model incorporating a common method latent factor but no constraints on the regression weights. Using the procedure by Meng et al. (1992), out of the 49 regression coefficients that had been compared between the models, only the association between integrity and abusive leadership turned out to significantly differ dependent on whether a common latent method factor had been regarded or not (*r*_common method latent factor_ = −0.95 vs. *r* = −0.71, *z* = 7.16, *p* < 0.001). Analyses incorporating ethical, transformational, and servant leader showed that common method variance was indeed a serious problem among these constructs, since as much as 47.6% of the variance was explained by a single underlying latent factor. Comparisons of correlated correlation coefficients between the models revealed only one significant difference concerning the relations among the ELW-D-dimensions (*r*_PO, CFS common method latent factor_ = 0.66 vs. *r*_PO, CFS_ = 0.83, *z* = 3.32, *p* < 0.001), and a significant difference of the correlations between transformational leadership and role clarification (*r*_common method latent factor_ = 0.48 vs. *r* = 0.79, *z* = 4.48, *p* < 0.001) as well as concern for sustainability (*r*_common method latent factor_ = 0.54 vs. *r* = 0.81, *z* = 4.14, *p* < 0.001). However, all of the relations between ELW-D-dimensions and servant leadership significantly differed as a function of either including or not including a common latent method factor (with *z*-values ranging between 12.60 and 28.68, all *p* < 0.001).

### Criterion-related validity

Assessing criterion-related validity, we first considered concurrent relations between the ELW-D and employees' work-related attitudes and behaviors. The overarching ethical-leadership-factor was associated with the outcomes considered in the way expected. All relations but with cynicism were positive and significant (Table 4). Aside from cynicism, the majority of subscales likewise exhibited significant positive correlations with the outcome variables. Using vector correlations (Hofmann and Jones, 2005) we tested whether ELW-D- and ELW-dimensions comparably related to these outcomes. We correlated the correlation coefficients between one dimension and all outcome variables obtained with the ELW-D with the corresponding coefficients reported for the ELW. The resulting correlation quantifies the similarity of associations within ELW-D and ELW. Except for the subscale concern for sustainability (*r* = 0.77, *p* < 0.01) all correlations ranged between *r* = 0.93 and *r* = 0.96 (all *p* < 0.001). Hence, across both measures dimensions are similarly associated with outcome variables.

Table 4**Correlation matrix of study variables in sub-sample 2**.

**Table:** 

	***M***	***SD***	**(1)**	**(2)**	**(3)**	**(4)**	**(5)**	**(6)**	**(7)**	**(8)**	**(9)**	**(10)**	**(11)**
(1) PO	3.45	0.90											
(2) PS	3.48	0.70	0.70^***^										
(3) F	3.79	0.96	0.55^***^	0.51^***^									
(4) RC	3.61	0.87	0.47^***^	0.45^***^	0.35^***^								
(5) I	3.64	0.92	0.68^***^	0.53^***^	0.66^***^	0.63^***^							
(6) CFS	2.91	1.01	0.53^***^	0.30^**^	0.15	0.45^***^	0.39^***^						
(7) EG	3.08	0.85	0.49^***^	0.47^***^	0.29^**^	0.68^***^	0.55^***^	0.53^***^					
(8) ELW-D	3.44	0.66	0.85^***^	0.76^***^	0.69^***^	0.75^***^	0.84^***^	0.60^***^	0.76^***^				
(9) ELS-D	3.21	0.72	0.78^***^	0.69^***^	0.50^***^	0.54^***^	0.65^***^	0.51^***^	0.65^***^	0.83^***^			
(10) Job satisfaction	3.58	1.05	0.44^***^	0.51^***^	0.43^***^	0.34^**^	0.43^***^	0.26^*^	0.44^***^	0.55^***^	0.44^***^		
(11) Satisfaction with the leader	3.39	0.97	0.68^***^	0.61^***^	0.60^***^	0.54^***^	0.65^***^	0.38^***^	0.49^***^	0.76^***^	0.73^***^	0.61^***^	
(12) Effectiveness of the leader	3.45	0.94	0.74^***^	0.62^***^	0.54^***^	0.66^***^	0.75^***^	0.48^***^	0.60^***^	0.83^***^	0.77^***^	0.53^***^	0.77^***^
(13) Organizational Commitment	3.34	0.93	0.45^***^	0.50^***^	0.33^**^	0.32^**^	0.38^***^	0.40^***^	0.49^***^	0.55^***^	0.52^***^	0.76^***^	0.60^***^
(14) Team commitment	3.25	0.96	0.39^***^	0.34^***^	0.21^*^	0.29^**^	0.26^**^	0.43^***^	0.38^***^	0.43^***^	0.40^***^	0.52^***^	0.41^***^
(15) Trust in the organization	4.20	1.00	0.58^***^	0.65^***^	0.52^***^	0.57^***^	0.65^***^	0.46^***^	0.60^***^	0.76^***^	0.70^***^	0.70^***^	0.74^***^
(16) Trust in the leader	4.63	1.03	0.74^***^	0.68^***^	0.74^***^	0.51^***^	0.83^***^	0.35^***^	0.45^***^	0.82^***^	0.74^***^	0.52^***^	0.79^***^
(17) Employee effectiveness	4.04	0.63	0.24^*^	0.34^**^	0.26^*^	0.26^*^	0.25^*^	0.16	0.16	0.31^**^	0.32^**^	0.43^***^	0.41^***^
(18) OCB	5.42	0.60	0.19^†^	0.27^**^	0.34^***^	0.19^†^	0.16	0.21^*^	0.11	0.28^**^	0.27^**^	0.32^**^	0.29^**^
(19) Employee cynicism	2.29	0.79	−0.50^***^	−0.57^***^	−0.65^***^	−0.37^***^	−0.49^***^	−0.22^*^	−0.26^*^	−0.59^***^	−0.44^***^	−0.58^***^	−0.57^***^
(20) Psychological safety	3.75	0.71	0.46^***^	0.58^***^	0.64^***^	0.37^***^	0.49^***^	0.18^†^	0.19^†^	0.56^***^	0.48^***^	0.48^***^	0.59^***^
(21) Group cohesiveness	4.45	1.06	0.48^***^	0.54^***^	0.59^***^	0.30^**^	0.40^***^	0.26^*^	0.20^*^	0.53^***^	0.45^***^	0.48^***^	0.59^***^
(22) Job clarity	4.01	0.70	0.13	0.23^*^	0.18^†^	0.46^***^	0.18^†^	0.07	0.12	0.25^*^	0.20^†^	0.27^**^	0.30^**^
(23) Extra effort	3.29	1.09	0.49^***^	0.52^***^	0.26^*^	0.55^***^	0.41^***^	0.33^**^	0.48^***^	0.58^***^	0.56^***^	0.43^***^	0.49^***^

**Table:** 

	**(12)**	**(13)**	**(14)**	**(15)**	**(16)**	**(17)**	**(18)**	**(19)**	**(20)**	**(21)**	**(22)**
(1) PO											
(2) PS											
(3) F											
(4) RC											
(5) I											
(6) CFS											
(7) EG											
(8) ELW-D											
(9) ELS-D											
(10) Job satisfaction											
(11) Satisfaction with the leader											
(12) Effectiveness of the leader											
(13) Organizational Commitment	0.52^***^										
(14) Team commitment	0.47^***^	0.72^***^									
(15) Trust in the organization	0.78^***^	0.74^***^	0.56^***^								
(16) Trust in the leader	0.80^***^	0.47^***^	0.33^**^	0.75^***^							
(17) Employee effectiveness	0.39^***^	0.41^***^	0.35^***^	0.42^***^	0.39^***^						
(18) OCB	0.32^**^	0.34^**^	0.32^**^	0.44^***^	0.33^**^	0.41^***^					
(19) Employee cynicism	−0.56^***^	−0.49^***^	−0.48^***^	−0.68^***^	−0.64^***^	−0.42^***^	−0.41^***^				
(20) Psychological safety	0.53^***^	0.39^***^	0.40^***^	0.65^***^	0.66^***^	0.34^***^	0.44^***^	−0.81^***^			
(21) Group cohesiveness	0.51^***^	0.45^***^	0.44^***^	0.63^***^	0.58^***^	0.33^**^	0.48^***^	−0.79^***^	0.82^***^		
(22) Job clarity	0.45^***^	0.28^**^	0.40^***^	0.37^***^	0.28^**^	0.50^***^	0.44^***^	−0.33^**^	0.37^***^	0.30^**^	
(23) Extra effort	0.63^***^	0.50^***^	0.46^***^	0.59^***^	0.43^***^	0.41^***^	0.30^**^	−0.41^***^	0.38^***^	0.40^***^	0.42^***^

*N = 100. PO: people orientation; PS: power sharing; F: fairness; RC: role clarification; I: integrity; CFS: concern for sustainability; EG: ethical guidance; ELW-D: Ethical leadership at Work questionnaire German version; ELS-D: Ethical Leadership Scale German version*.

*** *p < 0.000*;

** *p < 0.01*;

* *p < 0.05*;

† *p < 0.10*.

To consider incremental validity of the ELW-D, we finally tested how strongly its dimensions contribute to outcomes over and above the ELS-D. Like Kalshoven et al. (2011a), we analyzed whether the seven ELW-D-dimensions account for variance increments in trust in the leader, leader effectiveness, employee effectiveness, and OCB over and above the ELS-D. Additionally, we extended the analyses to outcomes that Kalshoven et al. (2011a) either included in correlational analyses (job satisfaction, satisfaction with the leader, organizational commitment, team commitment, trust in the organization, cynicism) or that have not yet been analyzed as outcomes of ethical leadership (psychological safety, group cohesiveness, job clarity, and extra effort). Linear relationships among the dimensions could have distorted the results of our analyses, but multicollinearity did not occur. Results of hierarchical regression analyses are depicted in Table 5. Varying subsets of the seven dimensions accounted for variance increments in all outcomes but employee effectiveness and team commitment over and above the ELS-D. Incremental validity of the ELW-D-dimensions hence holds for the majority of work-related attitudes and employee behaviors.

**Table 5:** **Incremental validity of the ELW-D subscales over and above the ELS-D**.

**Variable**	***B***	***SE***	**β**	***t***	***B***	***SE***	**β**	***t***	***B***	***SE***	**β**	***t***	***B***	***SE***	**β**	***t***
	**Job satisfaction**				**Satisfaction with the leader**				**Effectiveness of the leader**				**Organizational commitment**			
**STEP 1**																
ELS-D	−0.13	0.24	−0.09	−0.54	0.54	0.16	0.40	3.32^**^	0.40	0.14	0.31	2.99^**^	0.19	0.21	0.14	0.91
**STEP 2**																
PO	0.02	0.19	0.02	0.11	0.13	0.13	0.12	0.95	0.18	0.11	0.17	1.64	−0.09	0.17	−0.09	−0.56
PS	0.46	0.20	0.31	2.36^*^	0.10	0.14	0.07	0.71	0.04	0.11	0.03	0.39	0.41	0.17	0.31	2.38^*^
F	0.25	0.13	0.22	1.86^†^	0.22	0.09	0.22	2.46^*^	0.03	0.08	0.03	0.38	0.12	0.12	0.12	1.00
RC	−0.11	0.16	−0.09	−0.71	0.21	0.11	0.18	1.91^†^	0.24	0.09	0.22	2.71^**^	−0.17	0.14	−0.16	−1.27
I	0.04	0.17	0.04	0.24	0.05	0.12	0.05	0.45	0.25	0.10	0.24	2.53^*^	−0.03	0.15	−0.03	−0.17
CFS	0.03	0.12	0.03	0.23	0.00	0.08	0.00	0.01	0.03	0.07	0.03	0.40	0.18	0.10	0.19	1.75^†^
EG	0.38	0.17	0.31	2.28^*^	−0.09	0.12	−0.08	−0.77	0.00	0.10	0.00	−0.30	0.31	0.15	0.28	2.14^*^
*R*^2^				0.35^***^				0.64^***^				0.74^***^				0.38^***^
Δ *R*^2^				0.16^**^				0.11^**^				0.15^***^				0.11^*^
	**Team commitment**				**Trust in the organization**				**Trust in the leader**				**Employee effectiveness**			
**STEP 1**																
ELS-D	0.08	0.23	0.06	0.33	0.38	0.17	0.27	2.23^*^	0.38	0.12	0.27	3.23^**^	0.26	0.16	0.29	1.58
**STEP 2**																
PO	0.11	0.19	0.11	0.59	−0.28	0.14	−0.25	−2.01^*^	0.02	0.10	0.02	0.24	−0.18	0.13	−0.26	−1.36
PS	0.17	0.19	0.12	0.88	0.46	0.14	0.32	3.30^**^	0.27	0.10	0.18	2.75^**^	0.23	0.13	0.26	1.73^†^
F	0.07	0.13	0.07	0.51	0.14	0.09	0.13	1.48	0.25	0.70	0.23	3.79^***^	0.07	0.09	0.11	0.82
RC	0.00	0.15	0.00	−0.06	0.06	0.11	0.05	0.54	−0.02	0.08	−0.02	−0.26	0.13	0.11	0.18	1.22
I	−0.14	0.17	−0.14	−0.86	0.25	0.12	0.23	2.08^*^	0.53	0.09	0.47	6.20^***^	0.00	0.12	0.00	0.01
CFS	0.26	0.12	0.28	2.27^*^	0.17	0.08	0.17	2.03^*^	0.01	0.60	0.01	0.20	0.06	0.80	0.10	0.79
EG	0.17	0.17	0.15	1.01	0.12	0.12	0.10	1.03	−0.17	0.08	−0.14	−1.99^†^	−0.17	0.11	−0.23	−1.51
*R*^2^				0.26^**^				0.64^***^				0.83^***^				0.17^*^
Δ *R*^2^				0.10				0.16^***^				0.29^***^				0.07
	**OCB**				**Cynicism**				**Psychological safety**				**Group cohesiveness**			
**STEP 1**																
ELS-D	0.22	0.15	0.27	1.50	0.09	0.15	0.08	0.60	0.21	0.13	0.21	1.58	0.17	0.22	0.11	0.78
**STEP 2**																
PO	−0.21	0.12	−0.31	−1.68^†^	−0.01	0.13	−0.01	−0.07	−0.17	0.11	−0.22	−1.59	−0.04	0.17	−0.04	−0.25
PS	0.14	0.12	0.17	1.17	−0.40	0.13	−0.35	−3.12^**^	0.39	0.11	0.38	3.53^**^	0.50	0.18	0.34	2.83^**^
F	0.27	0.08	0.43	3.28^**^	−0.42	0.09	−0.51	−4.92^***^	0.35	0.07	0.47	4.68^***^	0.54	0.12	0.49	4.47^***^
RC	0.12	0.10	0.17	1.19	−0.13	0.10	−0.14	−1.28	0.17	0.09	0.24	1.91^†^	0.15	0.14	0.12	1.05
I	−0.17	0.11	−0.25	−1.54	0.03	0.11	0.03	0.26	0.01	0.10	0.01	0.09	−0.18	0.16	−0.16	−1.18
CFS	0.16	0.07	0.27	2.21^*^	−0.08	0.08	−0.11	−1.10	0.07	0.07	0.10	1.05	0.20	0.11	0.29	1.83^†^
EG	−0.17	0.11	−0.24	−1.61	0.13	0.11	0.14	1.21	−0.29	0.09	−0.34	−3.05^**^	−0.31	0.15	−0.25	−2.02^*^
*R*^2^				0.23^**^				0.52^***^				0.55^***^				0.47^***^
Δ *R*^2^				0.16^*^				0.33^***^				0.32^***^				0.27^***^
	**Job clarity**				**Extra effort**											
**STEP 1**																
ELS-D	0.18	0.16	0.19	1.12	0.39	0.23	0.26	1.69^†^								
**STEP 2**																
PO	−0.12	0.13	−0.16	−0.92	0.13	0.19	0.11	0.69								
PS	0.12	0.13	0.12	0.88	0.32	0.19	0.21	1.69^†^								
F	0.08	0.09	0.10	0.83	−0.11	0.13	−0.10	−0.88								
RC	0.61	0.11	0.76	5.76^***^	0.47	0.15	0.37	3.12^**^								
I	−0.16	0.12	−0.21	−1.34	−0.11	0.17	−0.10	−0.68								
CFS	−0.03	0.08	−0.05	−0.39	−0.04	0.11	−0.04	0.73								
EG	−0.32	0.11	−0.39	−2.80^**^	0.01	0.16	0.01	0.08								
*R*^2^				0.32^***^				0.44^***^								
Δ *R*^2^				0.29^***^				0.13^**^								

*N = 100. The Bs are taken from the last step of the regression. Tolerance and Variance Inflation Factor are > 0.02, respectively < 4. ELS-D: Ethical Leadership Scale German version; PO: people orientation; PS: power sharing; F: fairness; RC: role clarification; I: integrity; CFS: concern for sustainability; EG: ethical guidance*.

† *p < 0.10*,

* *p < 0.05*,

** *p < 0.01*,

*** *p < 0.001*.

## Discussion

The primary purpose of the present study was to validate a German version of the multi-dimensional ELW. In so doing, we replicated findings with the ELW, but also refined the analyses as we additionally intended to advance the nomological network of ethical leadership.

### Factor structure

In accordance with the ELW, the ELW-D consists of the dimensions people orientation, power sharing, fairness, integrity, role clarification, concern for sustainability, and ethical guidance. Fit indices attest the seven-factor solution an acceptable fit to the data, compared to a good fit reported for the ELW. Nested-model comparisons suggest the seven-factor solution to be the best fitting model among those tested. The factor structure holds in each of the three sub-samples and analyses of vector correlations support the accordance of ELW-D and ELW with regard to the associations between the seven dimensions. Considering the model with an overarching ethical-leadership-factor fit indices are ambiguous. Since the fit index AIC also stresses a better fit of the seven-factor solution, we commend future research to rather rely on ELW-D-dimensions.

### Convergent and discriminant validity

Supporting convergent validity of the scale, we found significant correlations among the ELW-D and construct-related leader behaviors. Both, observed correlations and analyses on the construct level show the ELW-D to converge least with abusive leadership. This finding gives evidence on the assumption that ethical and unethical leadership are rather two separate constructs than opposite ends of a continuum (see Brown and Treviño, 2006). Correlations further show the ELW-D to be highly convergent to the ELS-D. As CFAs suggest, though, instead of basing analyses on the overarching ELW-D-factor, the seven dimensions should be considered in research to come. Analyses of incremental validity stress the added value ELW-D-dimensions have in predicting outcomes over and above the ELS-D. Although the ELW-D encompasses core dimensions the ELS-D does not incorporate, measurement economy and specificity have to be carefully balanced when designing studies on ethical leadership. When the mere prevalence of ethical leadership is of interest, the use of the more economic ELS-D might suffice. If, however, studies aim at specifying how ethical leadership develops or operates and which behaviors are particularly effective or at providing detailed leadership feedback, the more thorough ELW-D might be the more suitable instrument.

Kalshoven et al. (2011a) related the ELW to age and gender and concluded discriminant validity based on their independence. We refined the analyses and found that despite being operationalizations of the same construct ELW-D and ELS-D are not or only marginally closer associated than ELW-D and servant or transformational leadership. Discriminant validity of the scale is hence restricted. Limitations of discriminant validity are of importance as alternatives to the core construct interpretations cannot be ruled out (Messick, 1995). Outcome relations we found might originate from the ELW-D-measure which seems to incorporate characteristics of transformational and servant leaders. Both have been shown to impact follower's performance, extra-role behavior, and work-related attitudes (e.g., Judge and Piccolo, 2004; Liden et al., 2008; Wang et al., 2011). Based on their descriptions, the question arises whether ethical, transformational, and servant leadership might excessively overlap on the conceptual level. Indeed, they share certain characteristics. However, ethical, transformational, and servant leaders also display a variety of unique behaviors that are not associated with the other leadership styles. Therefore, the three concepts may be considered independent (Brown et al., 2005; Brown and Treviño, 2006; Toor and Ofori, 2009; Kalshoven et al., 2011a; Den Hartog, 2015). Ethical leadership is set apart by its focus on the leaders' morality. Their moral management, that is their effort to guide followers ethically, to foster followers' ethical behavior, and to make ethics an explicit topic on the leadership agenda, is unique to ethical leaders (Treviño et al., 2000; Brown and Treviño, 2006; Toor and Ofori, 2009). Moreover, neither transformational nor servant leaders are concerned with the environment. Therefore, we doubt that our findings solely result from insufficient conceptual distinctiveness. Stressing deficiencies in the operationalization, Yukl et al. (2013) stated that by incorporating the dimensions role clarification, power sharing, people orientation, and concern for sustainability the ELW draws on facets which do not characterize ethical but traditional leaders. However, clarifying expectations and responsibilities, providing followers with voice, and being considerate is essential to ethical leadership (Brown and Treviño, 2006; Den Hartog and Belschak, 2012). Ethical leaders' care about sustainability has only recently been highlighted by qualitative research (Frisch and Huppenbauer, 2013), and characterizes them cross-culturally (Eisenbeiß and Brodbeck, 2013). However, rather than a misspecification of sub-dimensions, we assume the enormous degree of common source and common method variance to impact discriminant validity (Podsakoff et al., 2012). As followers' behaviors and attitudes are influenced by the way they perceive their leaders, they provide the best estimate of managerial conduct, and leaders' self-reports could have been affected by impression management (Brown and Treviño, 2006), we drew on followers to rate leadership styles. Within one survey, participants had to successively rate their leaders on the different scales. In so doing, they probably attempted to appear consistent across their answers (Podsakoff et al., 2012), yielding an undifferentiated, global judgment of leader behaviors. Additional analyses revealed that in our study as much as 47.6% of the variance in ethical, transformational, and servant leadership may be attributed to a common latent factor. Comparisons of correlated correlations coefficients corroborated that some of the correlations between ELW-D-dimensions and leadership styles have been inflated, while others have been deflated, and some have been left unaffected (see Podsakoff et al., 2012). As, however, the common latent factor may include variance resulting from true relationships between the constructs (Podsakoff et al., 2003), associations among the different leader behaviors urgently need to be considered in independent studies. Given that CFAs argue for a seven-factor solution, analyses should also draw on ELW-D-dimensions in investigating the relations with divergent constructs. For a final judgment of the psychometric properties of the ELW-D examination of discriminant validity needs to be resumed.

### Criterion-related validity

The ELW-D and its dimensions relate as assumed to the outcomes studied. Vector correlations acknowledge that association patterns are highly similar to those within the ELW. Since followers are the ones best aware of their attitudes, in validating both scales outcome measures mainly relied on self-reports (see Chan, 2009). As outcomes have been assessed together with ethical leadership in the same location using the same medium, common source and common method biases might have affected these associations. Nevertheless, correlation patterns with followers' effectiveness and OCB, which have been supervisor rated in the original work, are highly similar in the ELW-D and ELW. This finding suggests that biases of common source and common method variance possibly did not significantly affect the way dimensions related to outcome variables.

Despite the high correlations of the measures, in all outcomes but two ELW-D-dimensions significantly accounted for variance over and above the ELS-D. The newly included dimensions concern for sustainability, integrity, role clarification, and power sharing incrementally explained variance in followers' perception of work, their attitudes, and motivation. In leaders' effectiveness and employees' extra effort solely these dimensions accounted for variance increments. Nevertheless, concern for sustainability and integrity only contributed to the minority of outcomes. Leader's concern for the environment specifically accounted for follower's trust in the organization and OCB, and also seems to play a part in arousing followers' commitment and perceptions of group cohesiveness. As such, leaders who stimulate eco-friendly behaviors seem to particularly impact indicators of followers' attachment. This finding is in line with meta-analytic evidence revealing that CSR-measures most strongly relate to followers' commitment (Paruzel et al., 2015). The same was found when focusing on CSR-measures directed at eco-efficiency and the protection of the environment. Accordingly, employees more readily feel attached to their work, if sustainability is an organizational concern. Moreover, given the leader's broader scope which exceeds beyond immediate organizational affordances, also followers take on a broader perspective and proactively engage in contextual performance. Looking at the behaviors subsumed in the different dimensions, people orientation, power sharing, fairness, and role clarification are obviously more relevant in arousing leadership effectiveness, as well as follower's job satisfaction, extra effort, role clarity, effectiveness, or cynicism than concern for sustainability. Stimulating the recycling of items and materials or sympathizing with eco-friendly work processes does not necessarily ensure that leaders are effective in advocating organizational interests, eliciting performance beyond expectations, encouraging success, and reducing cynicism in work teams, or that followers know the best way and sequencing of activities to get tasks done, effectively complete their job, and are satisfied with job conditions or colleagues. The lack of incremental variance in these outcomes is thus hardly surprising. We suppose ELW-D-dimensions to differentially account for variance increments in various outcomes. Rather than exerting an impact on followers' core job performance, we assume that leader's concern for sustainability is more likely essential in evoking their pro-environmental workplace behavior. In support of this notion, pro-environmental leadership behaviors have been shown to positively affect a variety of green behaviors at work, such as environmentally friendly workplace behaviors and green advocacy within work groups (Robertson and Barling, 2013; Kim et al., 2014), environmentally responsible activities directed at the reduction of the company's ecological impact or the development of green processes (Graves et al., 2013) as well as employee green creativity and green product development performance (Chen and Chang, 2013), and followers' passion for the environment (Robertson and Barling, 2013). Accordingly, a reason for the small contribution of the sustainability dimension we found may be seen in the choice of outcomes that neglected the assessment of eco-friendly behaviors at work. In order to support the value of the sustainability dimension, green behaviors ought to be considered as outcomes of ethical leadership. In line with theorizing (e.g., Burke et al., 2007; Simons, 2008), we found integrity to primarily contribute to followers' trust. Earlier research revealed that through its impact on trust, integrity further relates to followers' commitment, job satisfaction, cynicism, and OCB (e.g., Kannan-Narasimhan and Lawrence, 2012; Moorman et al., 2013; Simons et al., 2015). In our study, we failed to provide evidence on the incremental effect of integrity on these outcomes. Findings by Cheng et al. (2015), though, rather assigned integrity a moderator function in predicting employees' commitment. In an American sample, they found integrity to boost the relation between perceived supervisor support and affective commitment while a compensatory effect was found for Chinese employees. Such interactive effects have not been considered here. Moreover, in assessing integrity, ELW-D items solely capture the leader's alignment of words and actions. However, word-action consistency is only one aspect of a leader's integrity (Martin et al., 2013). Besides, consistency of values and behavior, fairness, justice, honesty, and the guidance by strong personal moral values have been shown to characterize the construct across cultures (Martin et al., 2013). In Germany, the consistency of values and behavior, honesty, and a sense of responsibility toward others seem to predominantly qualify leadership integrity (Martin et al., 2013). Among the studies emphasizing the importance of integrity, several relied on such a broader conceptualization of the construct that at least added value-deed alignment (Craig and Gustafson, 1998; Kannan-Narasimhan and Lawrence, 2012; Moorman et al., 2013) or rather centered on interactional justice (Cheng et al., 2015). The fact that certain behaviors related to integrity are not included in the respective dimension of the ELW-D, and aspects like fairness, justice, honesty, responsibility toward others, or consideration are actually captured in distinct ELW-D-dimensions, may explain why we did not find integrity to account for variance increments in these outcomes. Also in the study by Kalshoven et al. (2011a), integrity only contributed to variance increments in trust in the leader and leader effectiveness. Consistent with earlier research (Resick et al., 2006; Palanski et al., 2015), we equally found integrity to contribute to variance increments in perceived leader effectiveness. Apart from the impact on followers' attitudes, research assigns integrity an important role for their ethical intentions (e.g., Peterson, 2004; White and Lean, 2008). As such, ethical intentions and ethical conduct of employees are important outcomes to consider in future research for which integrity might prove differentially effective. Power sharing, by contrast, significantly accounted for variance increments in a multitude of outcomes. Regarding followers' ideas in making decisions, delegating responsibilities, and permitting followers to play a central part in determining their performance goals, seem to be of special importance in impacting followers' attitudes and the way they perceive processes within teams. This dominance of the power sharing dimension is in line with intercultural research, attesting German employees very high participation expectations (Brodbeck and Frese, 2007). Accordingly, ELW-dimensions may be of differential significance dependent on the culture, norms, values, and orientations of the society they are studied in. Furthermore, the ELW-D extends the fairness dimension to work-related interactions and behavioral expressions of the leader's goal striving. Given this broader conceptualization, fairness incrementally contributes to followers' satisfaction with and trust in the leader, OCB, cynicism, psychological safety, and group cohesiveness. In certain outcomes, also ethical guidance and people orientation on which ELS-D items mainly focus significantly account for variance increments beyond the uni-dimensional scale. However, in none of the outcomes these dimensions had the greatest impact. In sum, the amount of variance additionally explained by the ELW-D ranges between 11 and as much as 33%. This clearly supports the added value of the multi-dimensional ELW-D. However, given the high relations with servant and transformational leadership, limiting analyses of incremental validity to the ELS-D is not justifiable. Rather, in drawing conclusions about the added value of the ELW-D, it is essential to additionally examine its contribution to outcome variables beyond servant and transformational leadership. As we assessed leadership behaviors and outcome variables in independent surveys, we were unable to conduct these analyses in the present study, though. Diverging from Kalshoven et al. (2011a), we found ELW-D-dimensions to not account for variance increments in employee effectiveness over and above the ELS. Since reliability of the employee effectiveness measure was low (α = 0.51), results have to be interpreted with caution. Besides, other subsets of dimensions than those reported by Kalshoven et al. (2011a) significantly add to the prediction of trust in the leader, leader effectiveness, and OCB, and findings concerning the additional outcomes partly deviate from our hypotheses: Unexpectedly, ethical guidance negatively related to psychological safety, cohesiveness, and job clarity. With regard to their teams, members might doubt whether colleagues really have good intentions or just pretend to behave ethically to avoid unpleasant consequences. That way, distrust may undermine the perception of safety and cohesiveness within teams. Besides, ethical guidelines and behavioral expectations might collide with standards on how to complete one's tasks. Such inconsistent demands may irritate followers and decrease job clarity. However, deviations from our assumptions may, again, stem from the study design. In multiple regression models systematic method variance of just one predictor can cause erroneous estimates of the predictors' effect and the amount of explained variance (Podsakoff et al., 2012). Whether its effect is attenuated or increased depends on the associations between the predictor and the outcome as well as the other predictors in a multiple regression. In order to more carefully examine the impact of ELW-D-dimensions, the inclusion of supervisor-rated, peer-rated, and objective criteria would be desirable.

In sum, the ELW-D shows psychometric properties comparable to those of the ELW. Whereas, we demonstrated convergent and criterion-related validity of the ELW-D, empirical evidence on the discriminant validity of both measures, the ELW-D and the ELW, still needs to be accumulated (see Den Hartog, 2015).

### Future research

Besides determining the prevalence of ethical leadership in Germany, research should identify its differential antecedents and mediators, in order to better understand the emergence and inner workings of ethical leadership behaviors. Whereas our findings are restricted to correlational self-reports, research on antecedents and mediators requires longitudinal designs. Many of the antecedents postulated concern personal characteristics of the leader like personality traits or personally held values (Brown and Treviño, 2006). Therefore, to further uncover determinants of ethical leadership and to understand how this kind of leadership develops, future studies should involve supervisors apart from employees. Moreover, as leadership demands interactions among leaders and followers, a dyadic approach to ethical leadership is indispensable. Cornelis et al. (2012), for example, showed that followers' needs can influence the emergence of ethical leadership. In Germany where supervisory boards, labor unions, and works committees are influential, an interactional approach may specifically focus on the impact these authorities have on the development and practice of ethical leadership.

Apart from validating the ELW-D this study aimed to identify further consequences of ethical leadership. Indeed, ethical leadership closely relates to follower's psychological safety, perceptions of group cohesiveness, and follower's extra effort. Associations with job clarity are also substantive. Though, we did not take into account that in varying contexts ethical leadership is probably differentially effective. Thus, in another line of research, the conditions under which ethical leadership is particularly powerful need to be identified. Conceivable moderators are followers' characteristics like their norms and values, or contextual factors like crises or the organizational climate. Although a climate for justice is said to promote the emergence of ethical leadership (Eisenbeiß and Giessner, 2012), it is also conceivable that ethical leadership only unfolds its full potential within organizations that value ethics. However, an ethical climate may equally be a bounding condition for moral persons to demonstrate moral management. Therefore, future research additionally needs to distinguish which factors contribute to the emergence and practice of ethical leadership and which facilitate its effectiveness.

Kalshoven et al. (2011a) first included a leader's concern for sustainability into their measure of ethical leadership. They called for further development of this scale and for items on a leader's concern for the society. Recently Frisch and Huppenbauer (2013) determined how ethical leaders behave toward various stakeholders. Based on their work, items on interactions with suppliers, owners, customers, the community, or the environment may be developed. As leaders who are fair, kind, and honest toward followers, but betray suppliers would not be considered ethical (Frisch and Huppenbauer, 2013), a stakeholder approach which considers the congruency of behavior between various target groups helps to develop the construct of ethical leadership.

Although the present study related ethical leadership to additional leadership behaviors and consequences, to more clearly specify its nomological network ethical leadership needs to be studied together with constructs like organizational justice, moral philosophies, or fair interpersonal treatment. Whether ethical leadership can be distinguished from these concepts is an important question to answer.

### Practical implications

The availability of a sound multi-dimensional German measure of ethical leadership also entails implications for organizations. Since followers prefer working for organizations which highlight ethics (Keith et al., 2003), the promotion of ethical leadership is an important target for practitioners. Given that ethical leadership can presumably be trained (Brown and Treviño, 2006), the multi-dimensional ELW-D can be used to detect concrete actions and specific ethical leader behaviors which might need more training in order to fully exploit the ethical leadership potential. Individually tailored trainings can be developed that focus on those distinct dimensions that need further development. Besides, training can also be designed with respect to the consequences of ethical leadership. As different dimensions differentially relate to indicators of leader effectiveness, training may equally concentrate on the practice of those behaviors that particularly impact the outcomes of interest. In either case highly efficient trainings result.

With the multi-faceted ELW-D detailed knowledge of the antecedents of ethical leadership can be accumulated to promote its development in German companies. If, for instance, situational variables like the climate within organizations foster the development of ethical leadership, companies might encourage its emergence through those means of organizational development (e.g., teambuilding) that create the beneficial climate. As ethical leaders, in turn, boost followers' ethical conduct (Mayer et al., 2009; Schaubroeck et al., 2012) and thus the ethical climate within teams, an upward spiral may result. If situational antecedents on the industrial or intra-organizational level which contribute to the development and preservation of ethical leadership (Eisenbeiß and Giessner, 2012) differentially relate to certain dimension of ethical leadership, means of organizational development may also be tailored to specifically stimulate those dimensions of ethical leadership, which are currently underdeveloped within an organization or management level. Thus, efforts of organizational development may also be conducted with a straighter goal-directedness, rendering them more efficient. Apart from the organizational context personality variables drive managers to lead ethically. Initial evidence suggests that ethical leaders are high in moral reasoning, conscientiousness, and agreeableness, but low in neuroticism (Brown and Treviño, 2006; Kalshoven et al., 2011b). Accordingly, personality variables and the value system a person holds are issues in the recruitment and selection of managers.

## Conclusion

With the ELW-D knowledge of concrete ethical leadership behaviors, their antecedents, mechanisms, outcomes, and preservation can be advanced in German-speaking countries. While it helps scientists to further define the construct, practitioners may enhance ethical leadership and ethical conduct within organizations rendering them more attractive places to work.

## Author contributions

All three authors (BS, AN, GM) substantially contributed to the development of the present study, to the analysis and interpretation of data, as well as to drafting and revising the paper. All authors approved the present version of the manuscript and agree to be accountable for all aspects of the work.

### Conflict of interest statement

The authors declare that the research was conducted in the absence of any commercial or financial relationships that could be construed as a potential conflict of interest.
