# Supplementary material for: Validation of a German Version of the Ethical Leadership at Work Questionnaire by Kalshoven et al. (2011)
Source: Front Psychol. 2016 Mar 31;7:446. doi: 10.3389/fpsyg.2016.00446 (PMC4815677; doi:10.3389/fpsyg.2016.00446)
Supplement: Supplementary file 1 [file Table1.pdf]

Appendix

Items of the German Ethical Leadership at Work questionnaire (ELW-D)

Scales/ items
**Mitarbeiterorientierung**
Meine Führungskraft interessiert sich dafür, wie ich mich fühle und wie es mir geht.
Meine Führungskraft nimmt sich Zeit für persönliche Gespräche.
Meine Führungskraft achtet auf meine persönlichen Bedürfnisse.
Meine Führungskraft nimmt sich Zeit, um über Gefühle zu sprechen, die die Arbeit betreffen.
Meine Führungskraft macht sich aufrichtig um meine persönliche Entwicklung Gedanken.
Meine Führungskraft zeigt Verständnis wenn ich Probleme habe.
Meine Führungskraft kümmert sich um ihre/ seine Mitarbeiter.
**Teilung von Macht**
Meine Führungskraft erlaubt Mitarbeitern, kritische Entscheidungen mit zu beeinflussen.
Meine Führungskraft erlaubt anderen nicht, sich an Entscheidungen zu beteiligen. (r)
Meine Führungskraft holt von Mitarbeitern Rat bezüglich der Organisationsstrategie ein.
Meine Führungskraft überdenkt Entscheidungen auf Grundlage von Empfehlungen derer, die ihr/ ihm unterstellt sind.
Meine Führungskraft delegiert verantwortungsvolle Aufgaben an Mitarbeiter.
Meine Führungskraft gibt mir die Möglichkeit, bei der Bestimmung meiner Leistungsziele eine Schlüsselrolle zu spielen.
**Fairness**
Meine Führungskraft macht mich für Probleme verantwortlich, über die ich keine Kontrolle habe. (r)
Meine Führungskraft macht mich für Arbeitsvorgänge verantwortlich, über die ich keine Kontrolle habe. (r)
Meine Führungskraft macht mich für Dinge verantwortlich, die ich nicht verschuldet habe. (r)
Meine Führungskraft strebt auf Kosten anderer nach ihrem/ seinem eigenen Erfolg. (r)
Meine Führungskraft konzentriert sich hauptsächlich darauf, ihre/ seine eigenen Ziele zu erreichen. (r)
Meine Führungskraft manipuliert Mitarbeiter. (r)
**Rollenklarheit**
Meine Führungskraft gibt an, welche Leistung von jedem Gruppenmitglied erwartet wird.
Meine Führungskraft erklärt, was von jedem Gruppenmitglied erwartet wird.
Meine Führungskraft erklärt, was von mir und meinen Kollegen erwartet wird.
Meine Führungskraft macht Prioritäten deutlich.
Meine Führungskraft macht klar, wer für was verantwortlich ist.

(continued)

Scales/ items
**Integrität**
Meine Führungskraft hält ihre/ seine Versprechen.
Meiner Führungskraft kann vertraut werden, die Dinge zu tun, die sie/ er sagt.
Auf meine Führungskraft ist Verlass, dass sie/ er ihren/ seinen Verpflichtungen nachkommt.
Meine Führungskraft hält immer ihr/ sein Wort.
**Nachhaltigkeit**
Meine Führungskraft würde gerne auf umweltfreundliche Weise arbeiten.
Meine Führungskraft macht sich Gedanken über Nachhaltigkeit.
Meine Führungskraft regt zum Recyceln von Gegenständen und Materialien in unserer Abteilung an.
**Ethische Führung**
Meine Führungskraft erklärt integritätsbezogene Verhaltensnormen deutlich.
Meine Führungskraft erklärt, was von Mitarbeitern hinsichtlich integeren Verhaltens erwartet wird.
Meine Führungskraft verdeutlicht Integritäts-Leitlinien.
Meine Führungskraft stellt sicher, dass Mitarbeiter die Integritäts-Leitlinien befolgen.
Meine Führungskraft macht deutlich, welche Konsequenzen bei unethischem Verhalten von mir und meinen Kollegen zu erwarten sind.
Meine Führungskraft regt die Diskussion über Integritäts-Aspekte unter den Mitarbeitern an.
Meine Führungskraft würdigt Mitarbeiter, die sich den Integritäts-Leitlinien entsprechend verhalten.
